# Luteinizing Hormone Regulation of Inter-Organelle Communication and Fate of the Corpus Luteum

**DOI:** 10.3390/ijms22189972

**Published:** 2021-09-15

**Authors:** Emilia Przygrodzka, Michele R. Plewes, John S. Davis

**Affiliations:** 1 Olson Center for Women’s Health, Department of Obstetrics and Gynecology, University of Nebraska Medical Center, Nebraska Medical Center, Omaha, NE 68198-3255, USA; emilia.przygrodzka@unmc.edu (E.P.); michele.plewes@unmc.edu (M.R.P.); 2 Veterans Affairs Nebraska Western Iowa Health Care System, 4101 Woolworth Ave, Omaha, NE 68105, USA

**Keywords:** corpus luteum, LH, mitochondria, lipid droplets, autophagy, cholesterol, progesterone

## Abstract

The corpus luteum is an endocrine gland that synthesizes the steroid hormone progesterone. luteinizing hormone (LH) is a key luteotropic hormone that stimulates ovulation, luteal development, progesterone biosynthesis, and maintenance of the corpus luteum. Luteotropic and luteolytic factors precisely regulate luteal structure and function; yet, despite recent scientific progress within the past few years, the exact mechanisms remain largely unknown. In the present review, we summarize the recent progress towards understanding cellular changes induced by LH in steroidogenic luteal cells. Herein, we will focus on the effects of LH on inter-organelle communication and steroid biosynthesis, and how LH regulates key protein kinases (i.e., AMPK and MTOR) responsible for controlling steroidogenesis and autophagy in luteal cells.

## 1. Introduction

The ovary is a highly dynamic organ that undergoes some of the most dramatic structural and functional changes of any adult tissue. Reproductive potential and reproductive life span are determined by the ovarian follicles, which are the major endocrine and reproductive compartments of the ovary. The follicles are comprised of three essential cells: theca cells, granulosa cells, and the oocyte. Together, these cells interact in a synergistic manner to secrete sex steroids and protein hormones that contribute to oocyte maturation for successful fertilization.

Gonadotropin-releasing hormone (GnRH) is released in a pulsatile fashion from the hypothalamus, inducing the release of the gonadotropins, follicle stimulating hormone (FSH) and luteinizing hormone (LH), from the anterior pituitary gland. FSH acts directly on granulosa cells of the developing ovarian follicle to stimulate growth of the follicle, secretion of estrogen and expression of the LH receptor (LHCGR) [1,2,3]. LH stimulates androgen production by the theca cells of developing follicles and triggers ovulation when follicular maturation is complete [4]. During the ovulatory process, LH causes the final maturation and release of the ovum from preovulatory follicles and stimulates the differentiation of the theca and granulosa cells of the preovulatory follicle into the small and large steroidogenic cells of the corpus luteum, respectively [5,6]. Corpus luteum formation involves interactions between steroidogenic cells, microvascular cells, and immune cells, leading to remodeling of the follicle and intense angiogenesis required for luteal formation [4,7]. As a result, the newly formed corpus luteum prepares and sustains the reproductive tract for conception, implantation, and early pregnancy [8,9,10].

The corpus luteum is a transient endocrine gland that synthesizes peptide and steroid hormones and serves as the primary source of progesterone during estrous/menstrual cycles and early pregnancy [10,11]. Progesterone acting via nuclear receptors is important for ovulation, embryo development, and preparation of the uterus for implantation [12,13,14]. Removal of the corpus luteum during the first trimester of pregnancy leads to miscarriage, whereas inappropriate production of progesterone is one of the main causes of preterm pregnancy losses in human [15]. Moreover, in some species such as dog, sheep, pig, and cow the corpus luteum persists and is necessary for most of pregnancy [16,17,18]. Therefore, appropriate function of the corpus luteum is crucial for pregnancy establishment and maintenance. Luteotropic and luteolytic factors precisely regulate luteal structure and function; yet, despite recent scientific progress within the past few years, the exact intracellular mechanisms controlling luteal function remain largely unknown. LH is the key luteotropic hormone that stimulates ovulation, luteal development, and maintenance of the corpus luteum. This pituitary gonadotropin induces not only the differentiation of the follicular cells into steroidogenic luteal cells, but also further stimulates progesterone biosynthesis in luteal cells. LH binds to LHCGR, a G-protein coupled, transmembrane receptor, present mainly on the steroidogenic small luteal cells. Canonical signaling by the LHCGR involves stimulation of adenylyl cyclase leading to increases in intracellular cyclic adenosine monophosphate (cAMP), activation of protein kinase A (PKA) and phosphorylation of PKA substrates, and ultimately stimulation of steroidogenesis. LH can also activate phospholipase C, leading to an increase in intracellular calcium and activation of mitogen-activated protein kinases (MAPK) [19,20]. Another luteotrophic and/or antiluteolytic factor is prostaglandin E2 (PGE2) that acts through four distinct G protein–coupled receptors (GPCRs), termed EP1–4, which activate signaling mechanisms similar to those produced in response to LH. PGE2 exerts a luteotrophic functions in multiple species, including the cow, pig, mare, rabbit, dog, monkey, and human [21,22,23,24]. In a reproductive cycle the corpus luteum terminates progesterone production and regresses in the absence of fertilization. Regression is triggered by a lack of luteotropic support and prostaglandin F2α (PGF2α), a key luteolytic factor that is produced within the gland or by the endometrium in domestic farm animals [16,25,26]. Notably, the early corpus luteum is insensitive to the luteolytic actions of PGF2α, but acquires luteolytic sensitivity later in the luteal phase in a species-specific manner [27,28,29]. Recent reviews provide excellent coverage of additional molecules that contribute to the formation, maintenance and regression of the corpus luteum [30,31,32,33].

Progesterone plays a vital role in fertility. Expression and activation of the progesterone receptor (PGR) is required for ovulation [14]. Furthermore, progesterone action is crucial for preparation of the uterine environment for implantation, embryo development and regulation of the estrous cycle [10,11]. The steroidogenic acute regulatory protein (STAR) mediates the rapid steroidogenic response to hormones by facilitating the transport of cholesterol across the outer mitochondria membrane to the inner mitochondria membrane (IMM). The cholesterol side-chain cleavage enzyme (CYP11A1) located within the inner mitochondria membrane, mediates the initial and rate-limiting step in steroidogenesis, converting cholesterol to pregnenolone [34]. Pregnenolone exits the mitochondria and is converted to progesterone by the enzyme 3β-hydroxysteroid dehydrogenase (HSD3B) located in the endoplasmic reticulum (ER). In the male, progesterone in further metabolized into testosterone by the enzymes cytochrome P450 Family 17 subfamily A member 1 (CYP17A1), and 17β-hydroxysteroid dehydrogenases (HSD17B) in the smooth ER [35]. Although steroidogenic luteal cells can synthesize cholesterol de novo [36], the majority of the cholesterol in luteal cells comes from the blood in the form of lipoproteins (LDL and/or HDL) [36]. Lipoproteins are internalized either through receptor-mediated endocytic or selective cellular uptake, where cholesterol is sorted from lipoproteins within endosomes. Transport of dietary cholesterol from endocytic organelles to the ER is essential for cholesterol homoeostasis, but the mechanism and regulation of this transport in luteal cells remains poorly defined [34,37,38].

In the present review, we summarize the recent progress in LH-triggered metabolic events in luteal cells. We review the contribution of various organelles and inter-organelle communication as a fundamental platform for the maintenance of basic luteal cell functions. The review also focuses on the role of LH on the activity of two main kinases (i.e., AMPK and MTOR) responsible for regulating metabolism, as well as metabolic events triggered downstream of 5′-AMP-activated protein kinase (AMPK) and mechanistic target of rapamycin (MTOR) in luteal cells. Due to the narrow focus of this review, the authors apologize for the inability to include all of the excellent contributions by other scientists studying the formation and regression of the corpus luteum.

## 2. Molecular Changes Occurring during Corpus Luteum Formation

Luteinization is a complex process characterized with rapid proliferation of endothelial cells, and cessation of proliferation and hypertrophy of the steroidogenic cells. In studies using a rat model, ovulatory doses of LH inhibited proliferation of granulosa cells [39]. Increased staining for a marker of proliferation (Ki67) was found among endothelial cells but not steroidogenic cells in the newly developed primate corpus luteum [40]. Similar observations were reported during luteal formation in cows, pigs, and sheep [41,42,43]. LH/PKA signaling triggers post-translational modifications or induction of early response transcription factors including phosphorylation of cAMP regulatory element binding protein (CREB), reproductive homeobox 5 (RHOX5) and early growth response-1 (EGR1), which cause multiple morphological and molecular changes [44,45,46,47,48]. Among the most prominent molecular changes triggered during luteinization are increased expression of mRNA transcripts for proteases (e.g., PTX3; ADAMTS1; CTS; VNN2; MMP9), as well as angiogenic (e.g., VEGFA; ANGPT; NOS3), or proinflammatory factors (e.g., PTGS2), which are crucial for tissue remodeling and formation of new vessels [23,48,49,50,51,52]. Higher expression of gene transcripts for lipoprotein uptake and cholesterol mobilization as well as steroidogenesis and progesterone signaling (e.g., LDLR, SCARB1, NPC-1, STAR, PGR) was also determined among LH triggered changes during luteinization [23]. In contrast, expression of factors involved in estradiol synthesis (e.g., CYP19A1), gonadotropin receptors (FSHR, LHCGR) or cell cycle (e.g., CDK2, CDK1, CDKN2, MCM3, MCM5, MCM6, CCNA2) was found to be downregulated during corpus luteum formation. Interestingly, proteomic analysis of preovulatory follicles of pigs revealed abundance of proteins involved in cellular infiltration, endoplasmic stress responses and protein ubiquitination. In addition, most of proteins in the newly formed corpus luteum were associated with steroid metabolism, cell death and survival, free radical scavenging, and protein ubiquitination pathways [53]. More cell specific transcriptomic characteristics were provided by Romereim et al., who determined expression of genes in the ovarian follicle cells and luteal cells [54]. Transcripts enriched in small and large luteal cells indicated increased metabolism/synthesis of lipids and steroids (cholesterol, eicosanoid, sterol, terpenoid, fatty acids andlipid membranes), cellular proliferation and survival, cell maturation, inflammatory and immune response, angiogenesis and migration of endothelial cells, and cell-to-cell signaling [54]. Among the predicted functions that were exclusive to the small luteal cells were metabolism of phospholipids, peptides, and sterols as well as regulation of the concentration of adenosine 5′-triphosphate (ATP). In large luteal cells, specific functions were related to adhesion (binding of cells, growth of epithelial tissue, and quantity of connective tissue) and cytoskeletal dynamics (microtubules and cell branching), molecular transport, development of blood cells, production of reactive oxygen species, and cellular homeostasis [54].

## 3. Morphological Characteristic of Small and Large Luteal Cells

**Figure 1:**
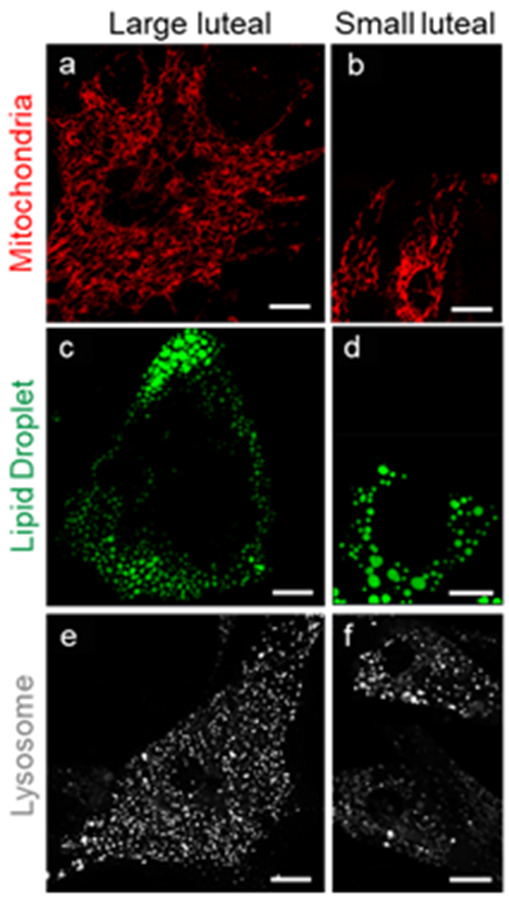
Organelle differences between steroidogenic large and small bovine luteal cells. Confocal microscopy was employed to visualize intracellular organelles in cultured bovine luteal cells. Representative micrograms of mitochondria ((**a**,**b**) MitoTracker); lipid droplets ((**c**,**d**) TopFluor Cholesterol);and lysosomes ((**e**,**f**) LysoTracker) in large and small luteal cells (left to right). Micron bar represents 10 µm.

**Figure 2:**
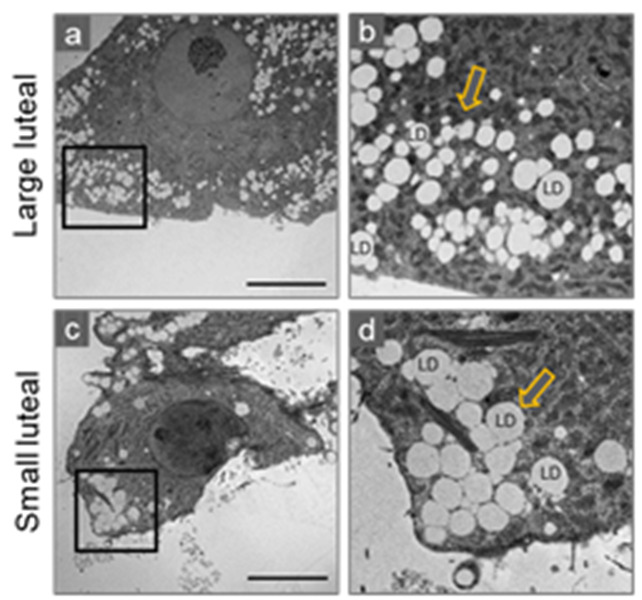
Distribution of lipid droplets in steroidogenic large and small bovine luteal cells. Transmission electron microscopy was employed to visualize intracellular lipid droplets (LD) in cultured bovine luteal cells. Representative micrograms of large (**a**,**b**) and small (**c**,**d**) luteal cells. Black box represents enlarged area (**b**,**d**). Arrows indicating LDs. Micron bar represents 10 µm.

In domestic farm animals like the cows, sheep and pigs, the small and large luteal cells are highly steroidogenic and differ based on the size, morphology, and function [55,56,57]. In the cow, the small luteal cells are 10–20 µm in diameter, possess a low cytoplasmic/nuclear ratio, and cytoplasm with mitochondria and numerous large lipid droplets. Large luteal cells are 30–40 µm in diameter, have a higher cytoplasmic/nuclear ratio, contain a well-developed, dense network of mitochondria and copious small lipid droplets dispersed through the cytoplasm (Figure 1 and Figure 2). Both small and large luteal cells are abundant in Golgi elements, granules, and ER or lysosomes [57]. Highly condensed chromatin is found at the nuclear periphery and in the central region of small luteal cells. However, in large luteal cells dispersed chromatin is found throughout the nucleus with condensed chromatin at the nuclear periphery [56,58,59]. The main functional differences between small and large luteal cells are that large luteal cells possess PGF2α receptor and produce 10- to 30-fold more progesterone than small luteal cells, whereas small luteal cells are highly responsive to treatment with LH and PKA activators [16,26,60,61].

## 4. Organelle Communication: Perspectives on Mitochondria, Endoplasmic Reticulum, Lipid Droplets, and Lysosomes in the Maintenance of Luteal Function

Intracellular organelles were once thought to function as individual units. However, recent progress in the understanding of membrane dynamics has revealed intracellular membrane compartments engage in extensive communication, either indirectly, or directly through membrane contacts, orchestrating dynamic communication between organelles that can be modulated to serve cellular requirements. Inter-organelle communication is fundamental for cell and tissue homeostasis as it regulates vital processes including calcium ions homeostasis, lipid and protein synthesis, metabolism, and cell death [62,63,64,65,66]. Inter-organelle communication also serves as platform for the control of signaling [67]. Here, we provide an overview of the mitochondria, ER, lipid droplets, and lysosomes in luteal cells and current information on inter-organelle communication and the functional roles of membrane contacts in ovarian steroidogenic cells.

### 4.1. Mitochondria

Mitochondria play a diverse role in cellular physiology from cell survival and metabolism, to initiating cell death mechanisms by producing free radicals and releasing apoptotic proteins [68]. These highly specialized organelles are bounded by two membranes, the outer and inner membranes, which function uniquely. The outer membrane contains protein-based pores regulating the passage of ions and molecules into the mitochondria, whereas the inner mitochondrial membrane is the active site for the electron transport chain and ATP production. Membrane integrity is crucial for mitochondrial function and depends on protein and phospholipid composition. Mitochondria are structurally dynamic organelles, continuously undergoing coordinated fission and fusion to sustain biological function [69]. In healthy cells, mitochondria maintain a highly regulated equilibrium between fusion and fission, forming either individual units or interconnected mitochondrial networks within the cell. The balance between mitochondrial fission and fusion often controls the overall fate of the cell [69,70]. In healthy cells, changes in mitochondrial morphology are under the control of fusion proteins [optic atrophy 1 (OPA1), mitofusin 1 (MFN1), and mitofusin 2 (MFN2)]; and fission proteins [dynamin 1 (DNM1), also known as dynamin related protein 1 (DRP1); dynamin 2 (DNM2); dynamin 1-like protein (DNM1L; commonly referred to as DRP1), mitochondrial fission factor (MFF), and fission 1 protein (FIS1)] [71,72,73,74]. These structural mitochondrial regulators are vital for proper development and tissue function. In vivo, Mfn1 or Mfn2 [75] or Opa1 [76] knockout mice are embryonic lethal as a result of insufficient mitochondrial fusion. Knockout of genes encoding Dnm2 [77] and Dnm1 [78] are also embryonic lethal; and DNM1L/DRP1 knockout mice die within two weeks of birth [79].

The dynamin family member DRP1 is a key mediator of outer mitochondrial fission. DRP1, a large dynamin GTPase, is recognized as the major player in mitochondrial fission in mammals [80], but the mechanism(s) by which DRP1 is regulated in steroidogenic tissues has only recently received attention. During mitochondrial fission, DRP1 is recruited to the mitochondria where it binds to its outer mitochondrial receptor, MFF, forming oligomeric complexes that surround to constrict and divide mitochondria. The regulation of DRP1 by post-translational modifications and differential phosphorylation is important for DRP1 translocation to mitochondria and induction of mitochondrial fission [81]. Phosphorylation of DRP1 at Ser616 by protein kinase C (PKC) [82] or cyclin dependent kinase (CDK) 1/cyclin B [83] results in activation and translocation of DRP1 from cytoplasmic compartment to mitochondrial membrane receptors. Moreover, phosphorylation of DRP1 on Ser616 mediates DRP1 interactions with other proteins and mitochondrial receptors to activate fission [84]. In contrast, phosphorylation of DRP1 at Ser637 within the GTPase effector domain (GED) domain inhibits DRP1 GTPase activity and translocation to mitochondria, thereby preventing mitochondrial fission [85,86,87]. The inactivation site of DRP1, Ser637, has a PKA consensus sequence and is widely accepted as a site for phosphorylation by PKA [86]. In the ovary, LH via PKA regulates the phosphorylation of DRP1 Ser637 in bovine luteal cells promoting optimal progesterone biosynthesis [88].

Genetic knockdown of DRP1 and MFF, but not fission protein FIS1, disrupts exogenous stimuli–induced mitochondrial fission and apoptosis in HeLa and MEF cells [89]. Recently, dynamin proteins have become novel targets to treat cardiovascular [90,91,92,93] and neurodegenerative diseases [94], as well as, cancer [95]. In small luteal cells genetic knockdown of MFF, the DRP1 mitochondria receptor, and a small molecule inhibitor of DRP1, Mdivi-1, increased both basal and LH-induced progesterone production [88]. These findings imply that LH may stabilize luteal mitochondria and promote cholesterol trafficking for steroidogenesis in bovine luteal cells via modulating the phosphorylation and activity of the mitochondrial fission protein DRP1.

Mitochondrial dynamin like GTPase (OPA1, also referred to as optic atrophy protein 1) is a mitochondrial dynamin related GTPase that is characterized as an inner mitochondria membrane fusion protein [96]. OPA1 is localized in the inner mitochondrial membrane and interacts with MFN1 and MFN2 to promote elongation by fusion of mitochondrial membranes. While previously described as a fusion protein, recent studies have emerged demonstrating that OPA1 has several additional important functions, such as stabilizing mitochondrial cristae, maintenance of mtDNA, and regulating the response of multiple tissues to apoptotic, necrotic, and atrophic stimuli [97].

Constitutive proteolytic processing of OPA1 by YME1 like 1 ATPase (YME1L) and/or OMA1 (zinc metallopeptidase) generates short (*S*-OPA1) and long (l-OPA1) isoforms that are released into the intermembrane space or tethered to the inner mitochondria membrane, respectively [98]. l-OPA1 contains a transmembrane domain and mediates mitochondrial fusion, while *S*-OPA1 takes on a soluble form and is involved in modulating mitochondrial energetics. Alterations to mitochondrial physiology, such as loss of mitochondrial membrane potential, ATP depletion or induction of apoptosis, leads to further proteolytically processing of l-OPA1 by OMA1 to generate the *S*-OPA1 isoform that is released into the intermembrane space. Regulation of OPA1 processing allows for alterations in the mitochondrial network through post-translational mechanisms that are more rapid than changes in gene expression [99]. In addition to sustaining mitochondrial homeostasis, this unique dynamin GTPase also serves as an A-kinase anchoring protein (AKAP). AKAPs act as scaffold proteins tethered to PKA and other signaling proteins such as phosphodiesterase, phosphatases and other protein kinases [100,101]. OPA1 has been shown to associate with lipid droplets, which are abundant in steroidogenic luteal cells [102]. In adipocytes, OPA1 associates with lipid droplets, serving as an AKAP, anchoring PKA for lipid hydrolysis. While the interaction between OPA1 and PKA on lipid droplets has been studied in adipocytes and adrenal cells [103,104], little is known about the role of OPA1 in ovary, specifically the corpus luteum. In small luteal cells, S-OPA1 is present on lipid droplets and interacts with PKA [105]. Interestingly, this interaction is amplified following acute LH-stimulation. Moreover, genetic knockdown of both, OPA1 and OMA1, using siRNA inhibits LH-induced progesterone synthesis, suggesting a critical role in steroid biosynthesis [106]. Additional studies are required to understand the processing, intra-cellular location, and role of OPA1 isoforms in steroidogenic cells.

### 4.2. Mitochondrial Associated Membranes

The mitochondria and ER are dynamic organelles that play a vital role in maintaining cellular homeostasis. Mitochondria are responsible for synthesizing ATP, maintaining intracellular of calcium ion (Ca^2+^) homeostasis and regulating apoptosis; while the ER is involved in protein folding, lipid metabolism and Ca^2+^ homeostasis [107,108,109,110]. Mitochondria and the ER continuously relay messages to maintain intracellular homeostasis and this communication is achieved by a physical association between the two organelles, forming a specialized microdomain termed mitochondria-associated membranes (MAMs) [111]. Tethering of mitochondria-ER membranes serves as a platform for cell signaling and scaffold for other proteins and regulatory factors [112,113,114].

Mitochondria-ER communication has been reported to increase upon hormone stimulation. Cholesterol synthesis occurs in the cytosol and ER through a multistep pathway beginning with acetyl-CoA. Newly synthesized cholesterol in the ER is rapidly transported to the plasma membrane by the secretory pathway and other organelles including the mitochondria and lipid droplets [111,115,116]. Mitochondria, however, are not part of the secretory pathway and, therefore, rely on lipid transport proteins to shuttle cholesterol from the ER. The AAA domain-containing protein 3 (ATAD3) is an essential mitochondrial protein that governs mitochondrial dynamics through the functional regulations of mitochondrial fission and fusion GTPases, DRP1 and OPA1, respectively. Of interest to luteal cells, ATAD3 has been identified as an essential component of the mitochondrial cholesterol transfer complex [117]. ATAD3A has the ability to assist with the transportation and metabolism of cholesterol by interacting with mitochondrial voltage-dependent anion channel (VDAC) and CYP11A1. The 67-kDa ATAD3 is anchored in the inner mitochondrial membrane and is enriched in at mitochondrial membrane contact sites [117]. Interestingly, in progesterone synthesizing cells, the 67-kDa long isoform of the ATPase family is also enriched in MAMs [118]. Although expression of ATAD3 is not hormonally regulated, ATAD3 localization participates in hormone-dependent steroidogenesis. Depletion of ATAD3 in mouse Leydig cells inhibits hormone-stimulated acute steroid biosynthesis, and this inhibition is recovered by treatment with 22R-hydroxycholesterol, a cell permeable metabolic cholesterol derivative [118]. This suggests ATAD3 plays a key role in the delivery of the substrate cholesterol into the mitochondria. A similar mechanism may occur in luteal cells, allowing direct delivery of substrate to the mitochondria for immediate progesterone production.

In steroidogenic cells, cholesterol is transported to the mitochondria by STAR, a transport protein that regulates cholesterol transfer within the mitochondria [37,119]. STAR is synthesized as a 37-kDa pre-protein that is further processed to a mature 30-kDa form. This is achieved by cleavage of an *N*-terminal mitochondrial import sequence. Recent studies have revealed voltage-dependent anion channel 2 (VDAC2) and translocase of outer membrane 22 (TOM22) are both involved in the regulation of STAR activity at the outer mitochondria membrane. In steroidogenic cells, STAR interacts with VDAC2 prior to its translocation to the mitochondrial matrix [117]. This interaction has been reported to occur at MAM sites and is a central location for initiating mitochondrial steroidogenesis [120]. In the absence of VDAC2, STAR is unable to interact with MAM-associated proteins and translocate into the mitochondria, halting steroid biosynthesis [120].

### 4.3. Mitochondria-Actin

Mitochondrial dynamics are dependent on interactions with microtubules and actin filaments, both components of the cellular cytoskeleton. Mitochondrial-cytoskeletal interactions have a well-established role in mitochondrial motility [121]. Actin cytoskeletal dynamics are regulated by the Rho GTPase family of proteins that consist of Rho, Rac, and Cdc42. During cell migration, Rho activation leads to the formation of stress fibers and retraction of the lagging edge of the cell during migration, whereas activation of Rac causes the formation of lamellipodia and membrane protrusions at the leading edge of the cell [122]. Members of the ADF/cofilin family of actin depolymerizing proteins are key regulators of actin dynamics and are considered essential for fundamental cellular processes [123]. In mammals, the ADF/cofilin family comprises three members: actin-depolymerizing factor (ADF, aka destrin), cofilin1, and cofilin2. Cofilin is regulated by cofilin binding molecules and differential phosphorylation and dephosphorylation. Phosphorylation of cofilin at Ser3 by LIM-kinases (LIMKs) and testicular protein kinases (TESKs) leads to inactivation whereas, dephosphorylation of cofilin by slingshot protein phosphatases (SSHs) leads to reactivation [123,124,125]. The signaling pathway initiated by hCG binding to the LHGCR leads to accumulation of intracellular cAMP and activation of PKA. In preovulatory granulosa cells, LH-induced PKA activation promotes rapid dephosphorylation of Ser3 of the actin-depolymerizing factor cofilin [126]. This dephosphorylation leads to rearrangement of the actin cytoskeleton and is required for progesterone biosynthesis [126,127].

### 4.4. Endoplasmic Reticulum (ER)

The ER is a large, dynamic organelle essential for maintaining cellular functions including Ca^2+^ storage, protein synthesis and lipid metabolism [128]. ER serves as a key site for protein synthesis of both secreted and integral membrane proteins, as well as a subpopulation of cytosolic proteins. Regulation of ER homeostasis plays vital roles in ovarian folliculogenesis, cumulus cells survival, cumulus–oocyte complex interactions and oocyte quality, luteal function, as well as pre-implantation embryo development [129,130,131,132].

ER homeostasis is sustained by ER chaperone proteins, glucose-regulated protein 78 (GRP78), heat shock protein 90 kDa beta member 1 (GRP94), calreticulin (CRT) and protein disulfide isomerase (PDI). ER stress occurs when the capacity of the ER to fold proteins becomes saturated, often as a result of impair protein glycosylation or disulfide bond formation, or by overexpression/mutations in proteins entering the secretory pathway [133,134]. In granulosa cells, increased follicular lipid accumulation has been reported to compromise ER function by activating ER stress pathways [135]. Activation of ER stress pathways attenuate hCG-stimulated PKA activation, and the expression and enzymatic activity of steroidogenic proteins, STAR and HSD3B, without affecting hCG-stimulated intracellular cAMP accumulation [136]. Moreover, induced ER stress reduces phosphorylation of PKA substrates responsible for cholesterol mobilization to the mitochondria leading to inhibition of progesterone biosynthesis. This increased activation of ER stress pathways has been shown to contribute to progesterone deficiency often associated with obesity [135,137]. ER stress has also been reported to contribute to the induction of profibrotic growth factors during ovarian fibrosis in polycystic ovary syndrome (PCOS) patients [138,139].

Recent studies demonstrated connection between metabolic hormones such as Visceral-adipose-tissue derived serine protease inhibitor (Vaspin) and ER homeostasis. Vaspin, also known as Serpin A12, is an adipokine that plays important roles in glucose metabolism and inflammation. Vaspin is a member of the serine protease family and binds to its receptor 78-kDa GRP78 ER chaperone. Like adiponectin and leptin, the level of vaspin are affected by gender, with significantly higher expression in females [140]. Recently, vaspin has been proposed to play a role in ovarian and luteal function. In the corpus luteum, vaspin and GRP78 expression are low during early luteal development and upregulated during the middle and late stages of the estrous cycles [141]. Interestingly, Kurowska et al. using immunohistochemistry, determined that vaspin and GRP78 were localized within the cytoplasm of both steroidogenic small and large luteal cells [141]. In vitro experiments reveal vaspin increases the expression of angiogenic and prolific genes, such as vascular endothelial growth factor (VEGFA), fibroblast growth factor 2 (FGF2), angiopoietin 1 (ANGPT1), VEGFA receptors (VEGFR1, VEGFR2), proliferating cells nuclear antigen (PCNA), and cyclin A, and decreases pro-apoptotic genes like caspase 3 and bcl-2-like protein 4 (BAX) in porcine luteal cells [141]. More research is required to determine how hormones and stress inducers impact the ER and ER-mitochondrial interactions in order to better understand the cellular control of steroidogenesis and luteal cell fate.

#### Lipid Droplets

Lipid droplets are unique organelles that serve as lipid reservoirs storing neutral lipids such as cholesterol esters and triglycerides [142,143,144]. Lipid droplets are coated with lipid droplet-associated proteins that embed within the surrounding phospholipid monolayer. These lipid droplet-associated proteins stabilize the droplet, interact with additional proteins that incorporate or remove lipids from the lipid droplet core, enable lipid droplets trafficking, and mediate association of lipid droplets with other organelles [145]. The perilipin (PLIN) proteins, designated PLIN1-5, are a family of lipid droplet coat proteins that are important for stabilizing lipid droplets structure and provide a platform for protein assembly on the lipid droplet surface. Although lipid droplets have been observed in nearly all tissues, lipid droplets have been most extensively studied in adipose cells, where they form large unilocular droplets [145]. Moreover, size, distribution, protein, and lipid content of lipid droplets can differ among various tissues [145]. Unraveling the dynamics and distinct characteristics of ovarian lipid droplets is key to fully understanding the role these lipid reservoirs in steroidogenesis and cell metabolism.

Ovarian luteal tissue is easily distinguished from other tissues due to the abundant lipid content and cytoplasmic lipid droplet [59]. Luteal droplets exist in all species examined to date including mice, rats, sheep, cattle, pigs, buffalo, rabbits, bats, non-human primates, and humans [146,147,148,149,150,151,152,153,154,155,156], and are abundant in both small and large luteal cells [59,157]. Bovine luteal cells contain higher levels of triglycerides than other tissues (cardiac, hepatic, & pulmonary) with the unsurprising exception of adipose tissue [59]. Additionally, the lipid droplets present in luteal cells are enriched with cholesterol esters and triglycerides that can be used to synthesize steroids in both humans and cattle [59,150,151,153]. The high triglyceride content of luteal tissue has unknown importance but could be the result of re-esterification of fatty acids liberated from cholesteryl esters during steroidogenesis to prevent fatty acid induced lipotoxicity. Interestingly, luteal lipid droplets are also enriched in key steroidogenic proteins required for optimal progesterone biosynthesis including STAR, vimentin, CYP11A1, and HSD3B. Moreover, acute stimulation with LH induces mobilization and/or increased expression of these key proteins to lipid droplets, suggesting a role of lipid droplets as signaling platforms for steroidogenesis [105].

Lipid droplets have a unique architecture enclosed by a phospholipid monolayer that is decorated by a specific set of proteins which forms the boundary between the aqueous cytosol and the hydrophobic inner lipid core. The PLIN family of lipid droplets coat proteins that are important for stabilizing lipid droplets structure and provide a platform for protein assembly on the lipid droplets surface. PLIN1 localizes to the surface of adipose tissue lipid droplets, however it is not clear whether PLIN1 binds at the surface or is embedded within the phospholipid monolayer. The activity of PLIN1 is regulated in a PKA-phosphorylation dependent manner, and its phosphorylation is known to rapidly initiate lipolysis. Adipocytes highly express PLIN1, a major lipid droplet binding protein, whereas PLIN1 is undetectable in lipid droplets of the bovine corpus luteum [59]. Luteal lipid droplets express PLIN2, PLIN3, PLIN5, hormone sensitive lipase, and 1-acylglycerol-3-phosphate *O*-acyltransferase, alpha beta hydrolase domain containing 5 and lower levels of adipose triglyceride lipase, and sterol *O*-acyltransferase proteins [59]. The presence of PLIN2, PLIN3, and PLIN5 suggest that luteal lipid droplets are metabolically active, oxidative, and mitochondrial-associated [145]. However, there appears to be a delicate balance between PLIN expression and luteal function. Preliminary studies from our laboratory indicate that overexpression of PLINs, 1, 2, or 3 change the size and distribution of luteal lipid droplets, detrimentally influencing acute LH-stimulated steroidogenesis [105].

Luteal lipid droplets are known to be regulated by luteal trophic hormones, such as LH, which mobilize lipid droplet cholesterol, and luteolytic hormones, such as PGF2α, which increase lipid droplet triglyceride content [156,158,159]. Hormone sensitive lipase (HSL) is an intracellular neutral lipase that hydrolyzes a variety of intracellular esters. It plays an essential role in lipid metabolism and is responsible for mediating the hydrolysis of di- and triacylglycerol, as well as cholesterol esters [160,161]. Hydrolysis of cholesterol esters by HSL in steroidogenic tissues provides substrate for the immediate synthesis of steroid hormones in adrenal glands, ovaries, and testis [58,162,163,164]. One of the unique features of HSL that differentiates it from other lipases is that its activity against triacylglycerol and cholesteryl ester substrates is regulated by reversible phosphorylation [160]. Structural studies have identified several amino acids and regions on HSL that are critical for regulating enzymatic activity. This has led to important insights into its function, including the hormone stimulated interactions with other key intracellular proteins, such as STAR [162] and lipid droplet-associated proteins, PLINs [165]. In luteal cells, LH/PKA signaling increases the hydrolytic activity of HSL by phosphorylation of a single site on Ser563, located within the regulatory module. Following cAMP/PKA dependent phosphorylation, HSL translocates to lipid droplets where it docks and interacts with other lipid droplet associated proteins. In adipocytes, the translocation of HSL to the lipid droplets occurs by virtue of PLIN1 localization to the surface of lipid droplets, physically interacting with HSL within the *N*-terminal region of PLIN1 to initiate the hydrolysis of cholesterol esters. This mechanism is not prominent in bovine luteal cells that express little PLIN1 [58,59]. Exactly how HSL associates with luteal lipid droplets is presently unknown.

Although HSL has broad substrate specificity, the kinetics of hydrolysis of neutral lipids and cholesterol esters vary based on fatty acid size and degree of unsaturation. Structurally, fatty acids consist of a carboxylic acid cap, a long aliphatic carbon chain, and hydrogen atoms along the length of the chain. In steroidogenic tissue, HSL more readily mobilizes fatty acids by hydrolysis as their chain length decrease (between 12–24 carbons) and degree of unsaturation increases [166,167]. The cholesteryl esters in bovine luteal lipid droplets are primarily (60%) mono- and polyunsaturated fatty acids evenly distributed among oleic acid (18:1), and the omega-3 and omega-6 20:4, 22:4 and 22:5 fatty acids [59]. This differs from ovine luteal cholesterol ester fatty acids which are predominantly palmitic acid [(16:0), 30.7%], oleic acid [(18:1) 22.3%], and linoleic acid [(18:2) 17.5%] [156]. Cholesteryl ester fatty acid content of rat luteal tissue had many similarities to those in bovine luteal lipid droplets, but contained more palmitic acid (16:0), and less oleic and arachidonic acids (18:1 and 20:4) content, and a reversed ratio of 18:0 to 18:1 fatty acids. The differences in fatty acid composition among these species could reflect species or diet effects [59,155,156,157]. Additional studies are needed to determine whether the fatty acids released upon HSL-induced cleavage of cholesterol esters enter luteal metabolic pathways or return to lipid droplets to be esterified into triglycerides.

### 4.5. Lipid Droplets and Mitochondria: Peri-Droplet Mitochondria

The notion that lipid droplets and mitochondria interact is well-supported. Luteal mitochondria also interact with lipid droplets at specific contact sites [58,59]. Proteomic analysis suggests that the luteal lipid droplets also interact with other key intracellular organelles involved in steroidogenesis and energy homeostasis, such as the ER, endosomes, and lysosomes [105]. Recently, a new subpopulation of lipid droplets interacting mitochondria have been described and may play a role in luteal steroidogenesis. Peri-droplet mitochondria have been reported in brown adipose tissue [168] and represent a segregated subpopulation of mitochondria that exhibit distinct composition, bioenergetics, and dynamics to support triglyceride synthesis. Peri-droplet mitochondria have higher levels of cytochrome c oxidase, ATP synthase, and super complex I + III assembly. Following stimulation of bovine luteal cells with analog of cAMP (8-BrcAMP), we observed an increase in proteins composing complex I and III (NDUA8, NDUA9, NDUBA, NDUS3, and NNRD), and ATP Synthase (ATPA, ATPB, ATPG, and ATP5H) [105] in isolated lipid droplets. Moreover, we reported an increased abundance of HADHA and HADHB, a mitochondrial trifunctional enzyme that catalyzes 75% of the reactions involved in the mitochondrial beta-oxidation pathway. This suggests following PKA activation, interactions between lipid droplets and mitochondria shift, promoting an intracellular environment for optimal steroidogenesis. One explanation for this rearrangement may be to prevent lipotoxicity. Fatty acids cleaved from cholesterol esters stored in lipid droplets may be quickly transported to a subpopulation of mitochondria capable of beta-oxidation during steroidogenesis.

### 4.6. Lysosomes

Lysosomes are the terminal degradative compartment of endocytosis, phagocytosis and autophagy [169]. Lysosomes regulate lipid metabolism through transcriptional as well as post-translational mechanisms. Lysosomes, once considered simple acidic organelles, are a hub for metabolic signaling regulated by different nutrient classes including amino acids and lipids. These dynamic organelles can integrate cellular signals and producing signal outputs. The lysosomal surface serves as a platform to assemble major signaling hubs, which relays multiple nutrient cues to the master growth regulators, MTOR, AMPK, and glycogen synthase kinase 3 beta (GSK3B) as reviewed further in Section 5.

Lysosomes play a dual role in both lipid transport and lipid biogenesis and integration of these two processes may play important roles in progesterone biosynthesis and luteal function [170,171]. One key role of lysosomes in steroidogenesis is mobilization of cholesterol from the lipoprotein complex. Cholesterol substrate for steroid biosynthesis in vivo is derived from internalized and degraded LDL and HDL. Drug inhibition of lysosomal functions using chloroquine, results in parallel inhibition of LDL degradation and progesterone production [172]. Cholesterol, the main substrate for progesterone synthesis, can also be provided by de novo synthesis in a multistep pathway from acetyl-CoA [37]. The reactions in the pathway occur in both the cytosol and ER. Newly synthesized cholesterol is rapidly transported from the ER to the plasma membrane and other organelles such as lipid droplets for storage or mitochondria for immediate steroid biosynthesis.

Lysosomes have been implicated in multiple events in the ovary, such as follicular and oocyte atresia, ovulation, steroidogenesis, and luteal regression [173,174,175]. Follicular atresia is an important process to ensure successful ovulation. Follicular atresia begins with the death of granulosa cells and finalizes with the degeneration of the oocyte. The pathway of granulosa cell death during follicular atresia depends on the state of energy metabolism related to follicular size. Small follicles result in changes in mitochondrial dynamics, whereas, lysosome function destabilizes in large follicles, resulting in major lysosomal rupture-induced necrosis.

Changes in lysosomes number and size, as well as enzyme activity have been implicated in late luteal regression in the porcine and bovine [176,177]. Lysosomes are important for luteal cell survival in addition to their well-established role in luteal regression. Mutations in TRPML1/Mcoln1, a lysosomal counter ion channel, reduced fertility in young and abolished fertility in old mice, despite normal mating activities [178]. Interestingly, corpus luteum formation was not impaired in old TRPML1/Mcoln1 mutant females; yet, progesterone biosynthesis was attenuated. This decrease in progesterone was accompanied by reduced expression of mitochondrial proteins, HSP60, and StAR, indicating impaired mitochondrial functions.

## 5. Metabolic Events Induced by LH in the Corpus Luteum: The Crosstalk of PKA/MTOR and AMPK Signaling in the Regulation of Metabolic Events in Luteal Cells

The maintenance of cell function requires utilization and metabolism of nutrients, which are an important source of proteins, lipids, amino acids, nucleotides, and energy. This is achieved through a network of well-synchronized cellular metabolic pathways that direct the utilization of various nutrients. Characterizing the principles underlying these cellular pathways can provide important insights into the understanding of cell function. Cellular metabolism is regulated by two main protein kinases i.e., AMPK and MTOR, which act in an antagonist manner. Both protein kinases are expressed in the granulosa and theca cells, oocytes and luteal cells [179,180,181,182,183]. For instance, AMPK activates catabolic processes such as autophagy, glucose transport, glycolysis, glycogen synthesis, or gluconeogenesis, whereas MTOR triggers anabolic processes including ribosome biogenesis and protein, nucleotide, fatty acid, and lipid synthesis [184,185]. The present section reviews how these key metabolic chaperones are regulated and the role of LH/PKA signaling on regulation of lipolysis, translation, and autophagy in luteal cells.

### 5.1. AMPK Attenuates Progesterone Production via Post-Translational Modifications of Enzymes Involved in Lipolysis in the Luteal Cells

AMPK is a trimeric protein composed of catalytic α subunit, scaffolding β subunit, and a regulatory γ subunit and is activated in response to reduced levels of ATP (elevated AMP/ATP ratio) [184]. Intracellular AMP/ADP directly bind to the regulatory γ subunit, inducing a conformational change that exposes an active phosphorylation site (Thr172) on the catalytic α subunit of AMPK (AMPKα) [184]. Phosphorylation of AMPKα at Thr172 is necessary for AMPK activation and can be triggered by upstream kinases, such as liver kinase B1 (LKB1) or in response to calcium flux via calcium/calmodulin dependent kinase kinase 2 (CAMKK2) [184,186]. Protein phosphatases (protein phosphatases 1 and 2) dephosphorylate AMPKα Thr172 leading to its inactivation. Moreover, PKA-dependent phosphorylation of AMPKα at Ser485 inhibits phosphorylation at Thr172, which limits AMPK activity [184].

AMPK regulates diverse metabolic events including inhibition of lipolysis and synthesis of fatty acids by phosphorylating HSL and acetyl-coA carboxylase alpha (ACACA) [184]. HSL interacts with lipid droplets and mediates the hydrolysis of cholesterol esters providing cholesterol substrate for immediate synthesis of steroid hormones [58,187]. ACACA catalyzes the carboxylation of acetyl-CoA to malonyl-CoA and is the rate-limiting enzyme in fatty acid synthesis. Phosphorylation of HSL at Ser565, a site specific for AMPK, prevents PKA-dependent phosphorylation and HSL activation [184,188]. Studies using bovine luteal cells showed that AMPK activity can be evoked by activating the PGF2α/CAMKK2 pathway leading to decreased progesterone production [179]. Moreover, activation of AMPK by AICAR (5-aminoimidazole-4-carboxamide-1-β-4-ribofuranoside) inhibits LH-stimulated progesterone production in bovine luteal cells [180,181]. Furthermore, AICAR-induced activation of AMPK triggers phosphorylation and inhibition of both ACACA (Ser79) and HSL (Ser565) in bovine luteal cells. Interestingly, the inhibitory effect of AICAR on LH-stimulated progesterone synthesis was ameliorated by incubating cells in the presence of 22-OH-cholesterol [181]. These findings suggest that AMPK acutely inhibits hormone-stimulated cholesterol mobilization in luteal cells, depriving cells of cholesterol the precursor for progesterone synthesis.

In contrast, treatment with LH increases the phosphorylation of HSL at Ser563 (activation site), a site specific for PKA, and decreases HSL phosphorylation at Ser565 (inactivation site). Additionally, LH prominently reduces phosphorylation of AMPKα at Thr172 (activation site), while simultaneously increasing the phosphorylation of AMPKα at Ser485 (inactivation site) [181]. These findings indicate that in luteal cells, AMPK, by virtue of its ability to inhibit HSL, acutely limits cholesterol mobilization from lipid droplets and inhibits steroid biosynthesis.

Metformin, an oral medication commonly used to treat type 2 diabetes, is a known activator of AMPK. Metformin is thought to activate AMPK by the inhibition of complex I of the electron transport chain leading to reductions in ATP production and increased content of AMP. The cellular bioavailability of metformin is controlled by membrane transporter proteins belonging to the solute carrier family (SLC) and multidrug resistant transporters of the superfamily of ATP-binding cassette (ABC) transporters [189]. In ovarian granulosa cells from multiple species, enhanced activity of AMPK in response to metformin decreased progesterone production by inhibiting transcription of steroidogenic genes, thus reducing the content of proteins involved in steroidogenesis [183,190,191]. In contrast, metformin did not affect progesterone production by luteal cells. Although, AICAR activation of AMPK prominently inhibited LH-stimulated progesterone production, it did not affect content of STAR, CYP11A1, and HSD3B in the luteal cells, pointing the importance of acute post-translational modifications mediated by AMPK in luteal steroidogenesis [181]. Interestingly, comparison of ovarian follicular cells to small and large luteal cells revealed reduced levels of transporters responsible for metformin uptake (SLC22A3) and increased transporters responsible for metformin secretion (ABCB1) in large and small luteal cells [54]. Thus, changes occurring during luteinization likely reduce the accumulation of metformin in luteal cells limiting its ability to activate AMPK. This may be beneficial for pregnant woman with diabetes who are treated with metformin.

### 5.2. MTOR Regulates Translation and Autophagy in the Luteal Cells

The MTOR is a conserved serine/threonine kinase, which forms two structurally and functionally distinct complexes i.e., MTORC1 containing MTOR, RPTOR (regulatory associated protein of MTOR complex 1 or raptor), MLST8 (MTOR associated protein, LST8 homolog), and PRAS40 (proline-rich AKT substrate of 40 kDa); and MTORC2 consisting of MTOR, RICTOR (RPTOR independent companion of MTOR complex 2), mLST8, and mSIN1 (also known as MAPK associated protein 1) [192,193]. MTORC1 regulates cell growth through modulating translation, in part by stimulating the phosphorylation of ribosomal protein S6 kinase beta-1 (S6K1) and eukaryotic translation initiation factor 4E binding protein (4EBP1), whereas MTORC2 regulates the phosphorylation of AKT on Ser473 and changes in the cytoskeletal that are involve in actin polymerization [194,195,196,197]. Both MTORC1 and MTORC2 are regulators of cellular metabolism. MTORC1 and S6K regulate metabolic enzymes directly, whereas mTORC2 evokes metabolic changes via AKT. S6K and AKT can also activate key metabolic transcriptional factors such as MYC proto-oncogene (MYC), hypoxia-inducible factor-1 (HIF-1), forkhead box (FOXO) transcription factors and sterol regulatory element binding transcription factor 1 (SREBF1) [185]. MTORC1 evokes various metabolic pathways including glucose metabolism via glycolysis and pentose phosphate pathway (PPP) as well as glutaminolysis and de novo lipogenesis in adipocytes, lymphocytes or cancer cells [185,193,198,199].

The activation of MTORC1 is dependent on nutrients and growth factors [185]. In the presence of nutrients such as amino acids (e.g., glutamine, arginine, leucine) MTORC1 translocates from the cytoplasm to the surface of lysosomes [198,200]. Activation of MTOR signaling is inhibited by the tuberous sclerosis complex (TSC), which consists of hamartin (TSC1) and tuberin (TSC2). TSC1 maintains the stability of the complex, whereas TSC2 functions as a GTPase and prevents the activation of MTOR by inhibition of the small G protein Ras homolog enriched in brain (RHEB) [201]. The activity of TSC2 is suppressed by growth factors or insulin via (PI3K)/AKT and/or MAPK1/3 signaling pathways leading to enhanced MTOR activity. In contrast, the activity of TSC2 can be evoked by AMPK and GSK3B resulting in a reduction in MTORC1 activity [202,203]. Studies on bovine luteal cells demonstrated that LH stimulates the phosphorylation and inactivation of GSK3B [180,204]. Additionally, LH/PKA pathway activates MTORC1 signaling reflected by increased activity of S6K1 and 4EBP1, proteins required for translation. LH mediated effects were independent on PI3K/AKT signaling since inhibition of PI3K/AKT signaling using wortmannin did not prevent the actions of LH on S6K1 phosphorylation. In contrast, treatment of luteal cells with insulin-like growth factor 1 (IGF-I) increased phosphorylation of the MTORC1 substrates S6K1 and 4EBP confirming that PI3K/AKT signaling is involved in S6K1 activity under basal conditions and in response to IGF-I but not to LH [180]. In rat granulosa cells, FSH stimulated PKA/MAPK signaling resulted in TSC2 phosphorylation leading to enhanced MTOR activity. In addition, inhibition of MTOR by treatment of granulosa cells with rapamycin decreased expression of cyclin D2, a critical factor regulating cell proliferation [205]. In human luteinized granulosa cells, rapamycin inhibited hCG-induced expression of steroidogenic genes (CYP11A1, HSD3B1 and STAR) as well as acute progesterone production [206]. Based on studies by Hou et al. [180] LH/PKA signaling stimulated MTOR activity in luteal cells enhancing phosphorylation of proteins related with translation but incubation with rapamycin did not affect basal or LH-stimulated progesterone production in bovine luteal cells. Thus, it seems that MTORC1 may have various roles in ovarian cells based on the state of cellular differentiation.

### 5.3. Autophagy

**Figure 3:**
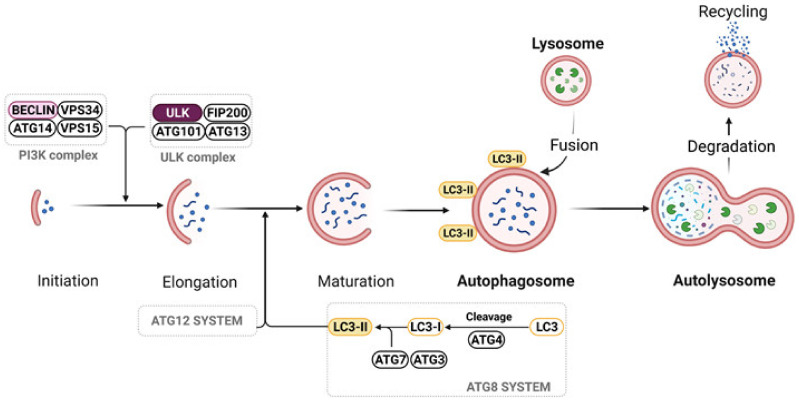
Process of autophagy. Autophagy is initiated by post-translational modifications in ULK1 or phosphatidylinositol-3-kinase (PI3K) complex. Autophagosomes elongation and maturation are catalyzed by ATG12 and ATG8 system, respectively. Formation of LC3-II occurs by proteolytic cleavage of pro-LC3 to LC3-I by ATG4, and then conjugation of LC3 family members to phosphatidylethanolamine (PE) on the surface of autophagosomes catalyzed by ATG7 and ATG3. Formed LC3B-II is a marker of autophagosomes. Mature autophagosomes fuse with lysosomes to form autolysosomes, which degrade cargo to amino acids, fatty acids or nucleotides that can be reused by cells. Created with BioRender.com. Adapted from “Autophagy In Cancer Pathways”, by BioRender.com (2021) (accessed on 1 September 2021). Retrieved from https://app.biorender.com/biorender-templates. accessed on 1 September 2021) [209].

Autophagy, so called ‘self-eating’, is a process during which cells destroy damage or used organelles to recycle inter-cellular content and building blocks required to maintain basic cellular functions [207]. Autophagy occurs at a basal level in normal functioning cells and can be also induced under certain conditions, such as starvation, low oxygen levels, and growth factor withdrawal, among others. Due to differences in cargo, autophagy is divided into microautophagy, chaperone-mediated autophagy, and macroautophagy. During macroautophagy targeted cytoplasmic constituents are isolated from the rest of the cell within a double-membraned vesicle known as an autophagosome. The content of the autophagosome is degraded when the autophagosome fuses with lysosomes (Figure 3). Various subtypes of macroautophagy have been identified based on the content of the autophagosome, such as mitophagy, ribophagy, lipophagy, and ER-phagy. In microautophagy, the lysosomal surface directly engulfs the cytoplasm, which is further degraded, whereas in chaperon mediated autophagy the material designated for degradation fuses with lysosomal membrane directly [207,208].

**Figure 4:**
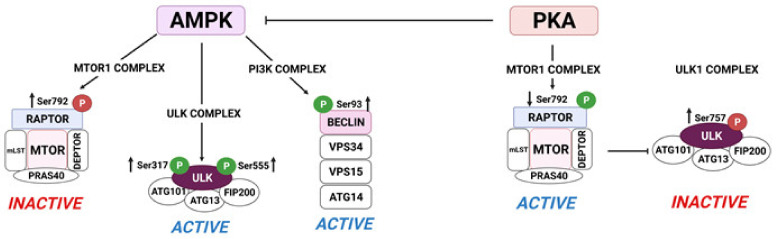
AMPK and PKA signaling oppositely regulate proteins crucial for autophagy initiation in bovine luteal cells. AMPK signaling stimulates autophagy initiation by (1) phosphorylation ULK1 at Ser317 and Ser555 leading to ULK1 activation; and (2) phosphorylation of RPTOR at Ser792 leading to MTOR inactivation. PKA signaling phosphorylates inhibits autophagy initiation by (1) phosphorylation of ULK1 at Ser757 and dephosphorylation of RPTOR at Ser792 allowing maintenance of MTOR activity. 5′-AMP-activated protein kinase (AMPK); protein kinase A (PKA); Unc-51-like kinase 1 (ULK1); regulatory associated protein of MTOR complex 1 (Raptor); MTOR associated protein, LST8 homolog (mLST8); proline-rich AKT substrate of 40 kDa (PRAS40); DEP domain containing MTOR interacting protein (DEPTOR); autophagy related 101 (ATG101); autophagy related 13 (ATG13); FAK family-interacting protein of 200 kDa (FIP200); autophagy related 14 (ATG14). Created with BioRender.com (accessed on 1 September 2021) [209]. “P” in the red circle represents phosphorylation site required for inactivation; “P” in the green circle means phosphorylation required for activation.

Autophagy is inversely regulated by the metabolic regulators, AMPK and MTOR. Activation of AMPK regulates autophagy at different levels including inactivation of MTOR via phosphorylation of RPTOR (Ser772 or Ser792) or TSC1/2 (Thr1227 or Ser1345) as well as direct activation of proteins related to autophagy induction and autophagosomes formation such as Unc-51-like kinase 1 (ULK1 Ser555 and Ser317) or (Ser93 or Ser15) (Figure 4). In addition, AMPK can also regulate the activity of transcription factor EB (TFEB) or FOXO3, nuclear transcription factors governing the expression of lysosomal and autophagic genes [208]. In contrast, MTOR inhibits autophagy by phosphorylation of ULK1 at Ser757, which disrupts the interaction between AMPK and ULK1 [210,211], thus preventing autophagosome formation [212,213].

In the ovary, autophagy has been associated with follicle development and atresia as well as luteal formation and regression [174,176,214,215,216,217,218,219]. Recently, Tang et al. (2020) found that HIF1α enhances autophagy in the rat luteinized granulosa cells [217]. HIF1α is highly expressed in the granulosa cells of preovulatory follicles in various species [220]. HIF1α upregulated BNIP3 (BCL2 interacting protein 3) expression in rat luteinized granulosa cells, which contributed to autophagy initiation by displacing Beclin1 from the Bcl2/Beclin complex and at the same time protecting cells from apoptosis [217]. In the rat corpus luteum, expression of autophagy related proteins increases simultaneously with decreases in Akt/MTOR signaling [218]. Moreover, treatment with 3-methyl adenine (3-MA), an inhibitor of PI3K, inhibited progesterone synthesis and decreased the number of lipid droplets in luteal cells. On the other hand, Beclin1 flox/flox conditional knockout mice had corpora lutea at pregnancy day (P) 8.5 suggesting that Beclin1 is not required for luteal formation. In pregnant dams, pregnancy began 4 days earlier than in wild type animals and had lower progesterone level after P13.5. Interestingly, females with one floxed allele and one null allele did not give birth, and resorption occurred between P5.5 and P14.5. In addition, Beclin1 conditional knockout corpora lutea have reduced lipid stores compared to wild-type luteal cells [221]. Therefore, it is possible that autophagy can be related with formation of lipid droplets in the luteal cells and therefore affecting progesterone synthesis.

Many independent studies demonstrate the occurrence of autophagy during luteal regression [176]. In cows, elevated expression of genes associated with autophagy (LC3α, LC3β, ATG3, and ATG7) and protein LC3B-II was observed in corpora lutea during the late luteal phase in comparison to corpora lutea of mid-luteal phase. These findings are correlated with studies showing enhanced activity of lysosomes and decreased expression of MTOR in the late corpus luteum [176]. In the pig, the expression of Beclin1 and LAMP1, proteins specific for autophagosomes and lysosomes, respectively, was highest in the corpus luteum during late luteal phase [215]. Our recent data showed elevated content of LC3B in the luteal tissue during induced luteolysis [222]. Moreover, Choi et al. demonstrated that after 24 h of PGF2α treatment, both LC3B and caspase 3 are increased in cultured rat luteal cells suggesting that autophagy can be associated with cell death during regression [214]. It is known that autophagy may lead to apoptosis and necroptosis [223] and it is possible that ‘autophagy mediated cell-death’ occurs during structural regression of the corpus luteum. A common element between autophagy and apoptosis is the interaction among Beclin1 and proteins of the BCL2 family. For example, Bcl-2 can bind with Beclin1 and this interaction can be regulated by posttranslational modifications of both proteins by phosphorylation, ubiquitination, or caspase-mediated cleavage [224,225]. Phosphorylation of Bcl-2 or Beclin1 disrupts the interaction between Beclin1 and Bcl-2 leading to autophagy [226,227]. This protein complex can be also disrupted by pro-apoptotic proteins such as BNIPS, Bad, Noxa, Puma or anti-apoptotic proteins such as tBid and Bad leading to autophagy initiation [226]. Recently, Beclin1 was found to be involved in the regulation of ferroptosis, a form of cell death triggered by lipid peroxidation after inhibition of the cystine/glutamate antiporter system xc-. This antiporter system is formed by two proteins: SLC7A11 (solute carrier family 7 member 11; the catalytic subunit) and SLC3A2 (solute carrier family 3 member 2; an anchoring protein) and plays a role in maintaining an intracellular redox homeostasis by importing cystine, which is used to synthesize the major antioxidant glutathione (GSH). Impairment of the system xc–-dependent antioxidant defense system results in oxidative injury and cell death. Phosphorylation of Beclin1 at S90/93/96 by AMPK increases binding to SLC7A11, while AMPK inhibition and mutation of S90/93/96 compromised the pro-ferroptotic function of Beclin1 in human cancer cell lines [228]. Therefore, understanding the factors that regulate the luteal Beclin1 interactome can be helpful to elucidate the role of Beclin1 and Beclin1 mediated autophagy in the maintenance of luteal function.

Recent studies demonstrate that AMPK and MTOR1 differentially regulate autophagic signaling pathways in bovine luteal cells [211,213]. In bovine small luteal cells stimulation of AMPK, a known negative regulator of luteal steroidogenesis, increased phosphorylation of RAPTOR (Ser792), which generates a docking site for inhibitory 14-3-3 proteins and is required for AMPK-mediated inhibition of MTOR1 [229]. In addition, treatment of luteal cells with an AMPK activator enhanced phosphorylation of Beclin1 (Ser93) and ULK1 (Ser317), two proteins necessary for autophagy initiation [222]. The effects of AMPK on phosphorylation of RPTOR, ULK1, and Beclin1 were abolished after pretreatment of luteal cells with inhibitor of AMPK (compound C) (Figure 4). Moreover, incubation with the AMPK activator AICAR increased the luteal content of LC3B-II, staining for autophagosomes and colocalization of lysosomes with autophagosomes confirming that activation of AMPK enhances autophagy in the luteal cells. In contrast, incubation of luteal cells with LH decreased activation of AMPKα which was correlated with reduced phosphorylation of RPTOR (Ser792). LH also increased phosphorylation of MTOR (Ser2448) and the MTOR substrates S6K (Thr389) and ULK (Ser757). Similar effects were observed after incubation of luteal cells with forskolin, an activator of adenylyl cyclase, whereas pretreatment with an inhibitor of PKA abolished LH-mediated effects on phosphorylation of AMPKα, ULK1 (Ser757), and RAPTOR (Ser792) [222] (Figure 4). Treatment with LH also rapidly decreased the content of LC3B-II and staining of autophagosomes thus confirming that LH inhibits signaling related with autophagy induction and autophagosome formation. Therefore, it is plausible that LH/PKA/MTOR and AMPK signaling differentially regulate autophagy in the luteal cells.

## 6. Summary

The cyclical development and regression of ovarian follicles and corpora lutea in the ovary represent one of the most fascinating endocrine-controlled, developmental biology systems in the adult. Proper systemic and local controls of these processes ensure fertility and fetal development, or resumption of the ovarian cycle in the absence of pregnancy. To understand the mechanisms of action of hormones and other factors that control ovarian steroidogenesis and cell fate it is imperative to appreciate the contribution of various intracellular organelles and potential inter-organelle communication as a fundamental platform for regulation and maintenance of basic luteal cell functions.

Understanding the intracellular mechanism of action of hormones and other factors requires knowledge of the contributions of two vital proteins kinases (i.e., AMPK and MTOR) to ovarian cellular metabolism, inter-organelle communication and steroidogenesis.

**Figure 5:**
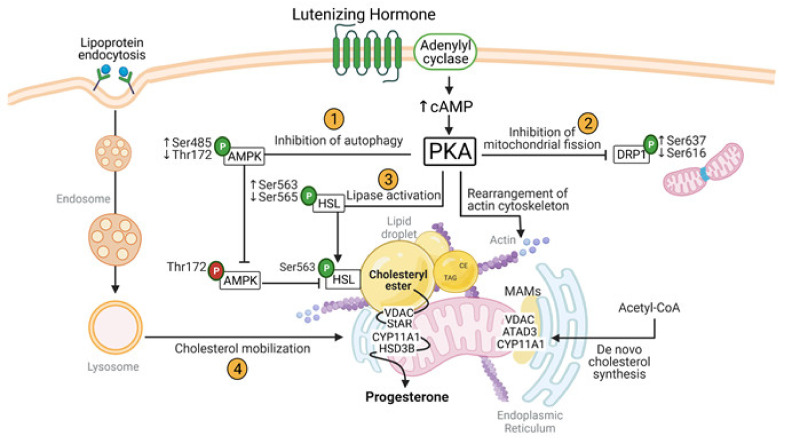
Cellular and metabolic changes induced by LH in steroidogenic luteal cells. Illustration of essential intracellular signals regulating inter-organelle communication provoked by LH/PKA signaling for optimal progesterone biosynthesis. Cyclic adenosine monophosphate (cAMP); protein kinase A (PKA); hormone sensitive lipase (HSL); steroidogenic acute regulatory protein (STAR); cholesterol side-chain cleavage enzyme (CYP11A1); 3β-hydroxysteroid dehydrogenase (HSD3B); voltage-dependent anion channel (VDAC); AAA domain-containing protein 3 (ATAD3); 5′-AMP-activated protein kinase (AMPK); mitochondrial associated membranes (MAMs); triglyceride (TAG); cholesteryl ester (CE). Created with BioRender.com [209]. “P” in the green circle means phosphorylation site regulated by LH; “P” in the red circle means phosphorylation site required for AMPK activation. Up and down arrows mean increased and decreased phosphorylation, respectively.

LH is a key luteotropic hormone that stimulates ovulation, luteal development, progesterone biosynthesis and maintenance of the corpus luteum. A summary of cellular and metabolic changes induced by LH in steroidogenic luteal cells is shown at Figure 5. LH via activation of its cognate cell surface receptor activates adenylyl cyclase, elevating cAMP and activating PKA. Intracellular signaling triggered by LH acutely (1) suppress autophagy, (2) stabilizes mitochondria, (3) activates lipolysis, and (4) stimulates cholesterol mobilization. Each of these events leading to steroidogenesis involves carefully orchestrated interactions among mitochondria, ER, lipid droplets, cytoskeletal fibers, lysosomes, and autophagosomes.

Over the last several years, considerable new research by many laboratories has identified several key factors and signaling mechanisms that play crucial roles in the cyclical development and regression of ovarian follicles and corpora lutea. These studies have contributed to significant enhancement of the basic knowledge underlying the mechanisms of hormone action, aiding the development of novel clinical approaches to improve female fertility or manage infertility-associated disorders. However, many questions remain unanswered:

- What is the repertoire of intracellular signaling events triggered by luteotropic and luteolytic agents? Where are these signals located in the cell and how do they affect cellular function?
- Can advanced cellular and organelle imaging techniques be used to improve our understanding of inter-organelle communication? Can this information be used to better understand cellular metabolism and control of steroidogenesis?
- Will analysis of the follicular and luteal proteome reveal actionable targets for improvement or control of fertility? Can we identify specific hormone-responsive protein kinases and their substrates in follicular and luteal cell types?
- How do hormones control post-translational changes other than phosphorylation (methylation, acetylation, ubiquitylation, etc.) and what is their contribution to ovarian function?
- What important cellular metabolic changes occur during the follicular to luteal transition? Can these be targeted to rescue or terminate luteal function?
- What are the intracellular metabolic changes induced by luteotropic and luteolytic agents? Can metabolites be identified that can regulate steroidogenesis and cellular fate?
